# Multiple Resistance Mechanisms to Tyrosine Kinase Inhibitors in EGFR Mutated Lung Adenocarcinoma: A Case Report Harboring EGFR Mutations, MET Amplification, and Squamous Cell Transformation

**DOI:** 10.3389/fonc.2021.674604

**Published:** 2021-06-11

**Authors:** Rossella Bruno, Marzia Del Re, Federico Cucchiara, Iacopo Petrini, Greta Alì, Stefania Crucitta, Agnese Proietti, Simona Valleggi, Antonio Chella, Romano Danesi, Gabriella Fontanini

**Affiliations:** ^1^ Unit of Pathological Anatomy, University Hospital of Pisa, Pisa, Italy; ^2^ Unit of Clinical Pharmacology and Pharmacogenetics, Department of Clinical and Experimental Medicine, University Hospital of Pisa, Pisa, Italy; ^3^ General Pathology, University of Pisa, Pisa, Italy; ^4^ Unit of Pneumology, University Hospital of Pisa, Pisa, Italy; ^5^ Department of Surgical, Medical, Molecular Pathology and Critical Area, University of Pisa, Pisa, Italy

**Keywords:** EGFR, lung adenocarcinoma, case report, multiple resistance mechanisms, MET amplification, squamous cell transformation

## Abstract

Resistance to EGFR tyrosin kinase inhibitors (TKI) inevitably occurs. Here it is reported the case of a young patient affected by lung adenocarcinoma harboring the L858R *EGFR* sensitive mutation. The patient developed multiple TKI resistance mechanisms: T790M *EGFR* resistance mutation, detected only on tumor cell-free DNA, squamous cell transformation and *MET* amplification, both detected on a tumor re-biopsy. The co-occurrence of squamous cell transformation and *de novo MET* amplification is an extremely rare event, and this case confirms how dynamic and heterogeneous can be the temporal and spatial tumor evolution under treatment pressure.

## Introduction

Patients with metastatic lung adenocarcinoma harboring epidermal growth factor receptor (*EGFR)* mutations benefit from tyrosine kinase inhibitors (TKI) treatment, but acquired resistance is inevitable (1, 2). Half of patients treated with first or second generation TKIs develops the *EGFR* secondary mutation T790M, being eligible for osimertinib second-line treatment (3). Besides T790M, pathways bypassing EGFR signaling and lineage transformation have been reported as resistance mechanisms, impacting on therapeutic assessment (4).

Here, it is presented a case with a rare coexistence of different resistance mechanisms: squamous cell transformation and MET amplification.

## Case Presentation

In August 2017, a 38-year-old man, former smoker, with cervical pain irradiated to the thorax left side underwent a computerized tomography (CT) scan evaluation, showing bone metastases in multiple vertebras and left pleural effusion. Thorax and abdomen CT scan, brain Magnetic Resonance Imaging (MRI), and 18-fluorodeoxyglucose positron emission tomography (PET) demonstrated a 3 cm tumor of the left lung inferior lobe with metastatic lymph nodes of the pulmonary hilum and homolateral mediastinum. Metastases were present in liver (3 lesions), right adrenal gland, multiple bone locations and brain (multiple sites). A diagnosis of cT1cN2M1c, stage IVc lung adenocarcinoma was made.

**Figure 1:**
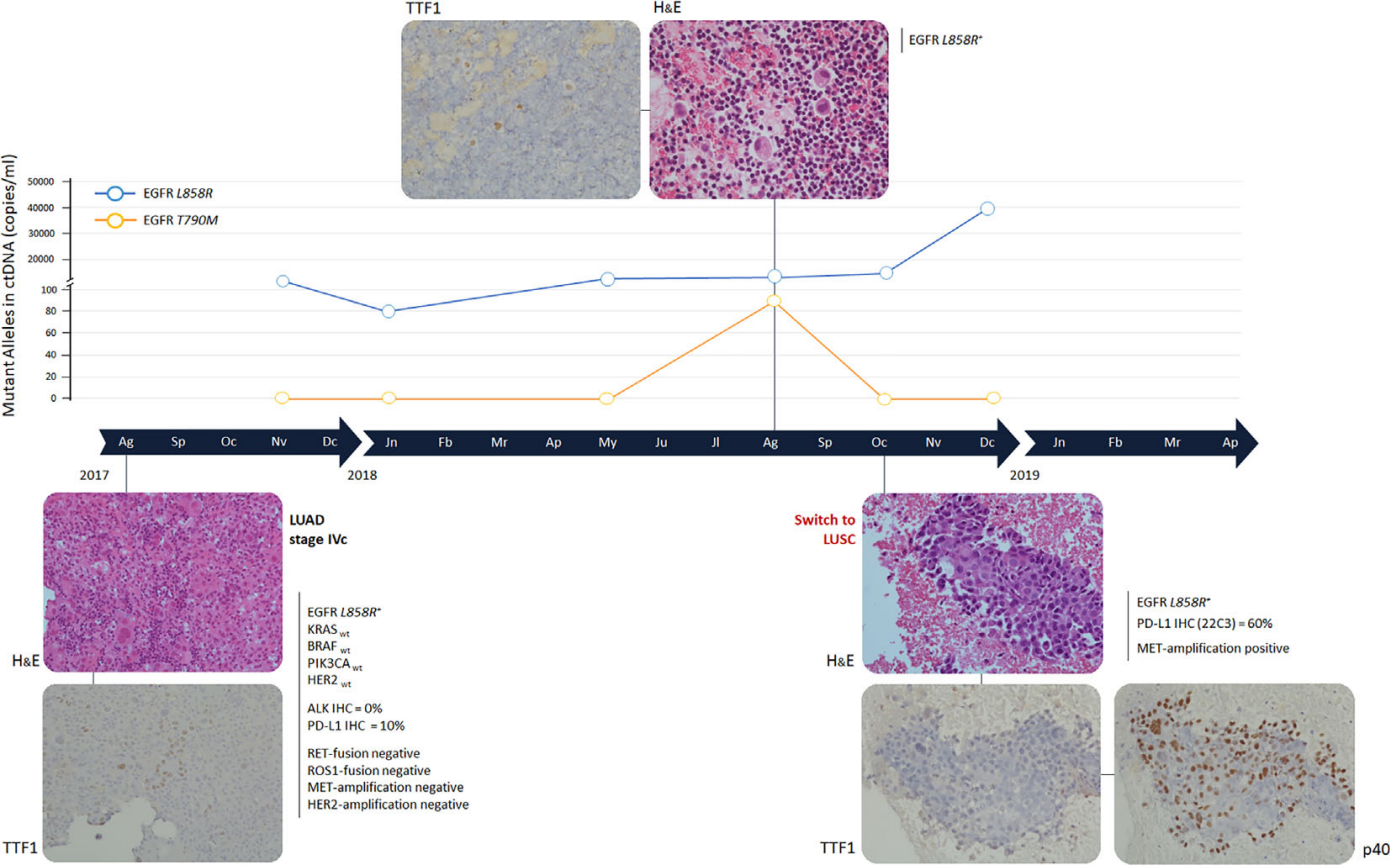
Timeline and histological transformation. H&E: Hematossil and Eosin staining (magnification 20X). Immunohistochemistry analyses were performed using mouse monoclonal antibodies anti TTF-1 and p40, clone 8G7G3/1 and clone BC28 respectively, on the Ventana Medical System (Roche). Immonuhistochemistry images have been reported with a 20X magnification. Gene mutational analysis was performed by Sequenom MassArray at baseline, *EGFR* mutational status was determined by digital droplet PCR at progression times. ALK and PD-L1 were evaluated by immunohistochemistry and gene fusions and amplifications by fluorescent *in situ* hybridization. LUAD, lung adenocarcinoma; LUSC, lung squamous-cell carcinoma.

The tumor was characterized by immunohistochemical (IHC) stain on cell-block from pleural effusion with anti-thyroid transcription factor (TTF-1) antibody (mouse monoclonal antibody; clone 8G7G3/1; Ventana Medical System - Roche, Monza, Italy), which showed a strong positive nuclear staining (**Figure 1**). The evaluation of the mutational status of *EGFR*, KRAS proto-oncogene, GTPase (*KRAS*), B-Raf proto-oncogene, serine/threonine kinase *(BRAF)*, phosphatidylinositol-4,5-bisphosphate 3-kinase catalytic subunit alpha *(PIK3CA)* and erb-b2 receptor tyrosine kinase 2 (*HER2*) genes was performed on the Sequenom MassArray platform using the Myriapod Lung Status kit (Diatech Pharmacogenetics, Jesi, Italy). Before DNA extraction and purification by the QIAamp DNA Mini Kit (Qiagen, Hilden, Germany), tissue enrichment for cancer cells was performed by manual macrodissection. The expression of CD274 molecule (PD-L1) and ALK receptor tyrosine kinase (ALK) was determined by IHC on the Ventana Medical System (Roche). The monoclonal primary antibody SP263 clone and the monoclonal primary antibody D5F3 clone were used for PD-L1 and ALK, respectively. The presence of gene fusions involving ROS proto-oncogene 1, receptor tyrosine kinase (*ROS1*) (Break Probe ROS1 (6q22) Kreatech - Leica Biosystems, Amsterdam, Netherlands) and ret proto-oncogene *(RET)* (Break Probe RET (10q11) Kreatech - Leica Biosystems) was evaluated by fluorescence *in situ* hybridization (FISH). FISH tests were executed also to analyze the amplification of MET proto-oncogene, receptor tyrosine kinase *(MET)* (Probes: LSI MET spectrum red and CEP7 spectrum green – Vysis - Abbott, Illinois, USA) and *HER2* (Probes: LSI HER2/neu spectrum orange and CEP17 spectrum green – Vysis, Abbott).

**Figure 2:**
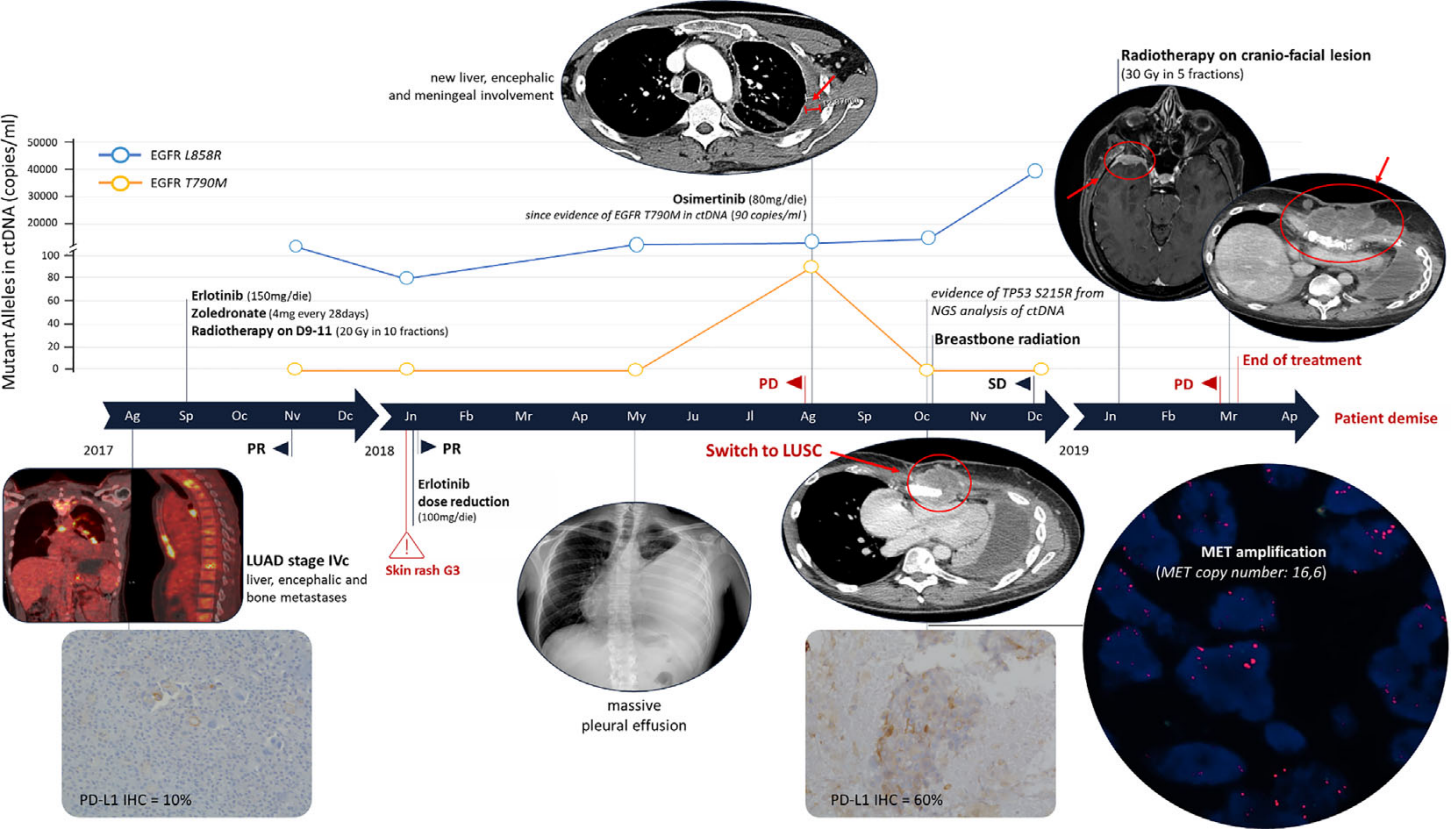
Clinical Timeline. The immunohistochemistry evaluation of PD-L1 was performed using a monoclonal primary antibody SP263 clone on the Ventana Medical System (Roche). MET amplification was evaluated by fluorescence *in situ* hybridization using the probes: LSI MET spectrum red and CEP7 spectrum green (Vysis – Abbott). PR, partial response; SD, stable disease; PD, progression disease; NGS, Next Generation Sequencing; LUAD, lung adenocarcinoma; LUSC, lung squamous-cell carcinoma.

The sensitive *EGFR* mutation L858R, within exon 21, was detected and 10% of cancer cells expressed PD-L1 (**Figure 2**). No other gene alterations were identified.

In September 2017, erlotinib treatment was started (150 mg/die) with zoledronate (4 mg every 28 days). In October, the patient completed the palliative radiotherapy on vertebral metastases of D9-11 (20 Gy in 10 fractions). After 2 months of treatment a partial response (PR) was observed with reduction of all disease localizations. *EGFR* mutational status in circulating tumor DNA (ctDNA) was evaluated every 3 months (**Figure 1**). ctDNA was purified from 3 ml of plasma by the QIAamp Circulating kit (Qiagen) and *EGFR* was analyzed by the digital droplet PCR (Bio-Rad, Hercules, California, USA) with the PrimePCR™ ddPCR™ Mutation Assay for human *EGFR*. In January 2018, erlotinib dosage was reduced to 100 mg because of a grade 3 skin rush of the face, truncus and extremities (National Cancer Institute – Common Terminology Criteria for Adverse Events). The CT scan confirmed the PR and the ctDNA analysis highlighted a decrease of L858R amount (**Figures 1** and **2**).

In May 2018, the patient presented with dyspnea and left hemithorax pain. A chest X-ray showed massive pleural effusion, an ultrasound-guided thoracentesis was performed. Tumor cells were not detected in the effusion, but ctDNA analysis revealed an increased L858R amount (**Figure 1**). The radiological response was maintained until August 2018, when a CT scan documented a pleural metastasis enlargement with pleural effusion. MRI of liver metastasis and brain demonstrated an enlargement of the left prerolandic metastasis and disease meningeal diffusion. An ultrasound-guided thoracentesis was performed with a diagnosis of adenocarcinoma metastasis, TTF1 positive (**Figure 1**). Cell-block from pleural effusion had a low percentage of tumor cells (<5%) and *EGFR* mutational status was evaluated by the same highly sensitive droplet PCR assay used for ctDNA analysis. On the pleural effusion only the L858R mutation was detected, whereas L858R sensitive and T790M resistance *EGFR* mutations were both detected on ctDNA. Therefore, in August 2018, the treatment was switched to osimertinib. However, the patient experienced thoracic pain and in October 2018 a CT scan demonstrated an oligoprogression of a sternal bone metastasis. A biopsy of the lesion showed a histological switch towards squamous cell carcinoma positive for tumor protein p63 (p40) (IHC was performed using a mouse monoclonal antibody, clone BC28, on the Ventana Medical System - Roche) and negative for TTF1, with PD-L1 expression observed in 60% of cancer cells (**Figure 2**). *MET* amplification (MET/CEP7: 5,8 - cut-off<2; MET copy number: 16,6 – cut-off<5) and *EGFR* L858R mutation were detected on the squamous lesion (**Figure 2**). The MRI showed the disappearance of brain metastases, and T790M mutation was no longer detectable in ctDNA, but the L858R amount increased. At the same time the ctDNA was analyzed also by Next Generation Sequencing (NGS) using the 56G Oncopanel (Diatech Pharmacogenetics) on a MiSeq platform (Illumina, San Diego, California, USA): the L858R mutation was confirmed (4.14 allele frequency) and a missense mutation, the S215R (4.73 allele frequency), within exon 6 of tumor protein p53 *(TP53)* gene was detected. The *TP53* mutation was confirmed both on the first pleural effusion and on the squamous lesion specimens. The patient underwent radiotherapy for the sternal metastasis, he experienced a pulmonary embolism with type I respiratory failure, and he received low molecular weight heparin with progressive recovery of the respiratory failure. Osimertinib was continued with a stable disease until December 2018. The ctDNA analysis found an increased L858R amount, in January 2019 the patient expedited a severe headache with retro-orbital pain and a skull CT scan showed a large secondary cranio-facial lesion, treated with radiotherapy (30Gy, 5 fractions). In March 2019, the patient showed neurologic and clinical deterioration without radiologic evidence of encephalic disease progression. A CT scan demonstrated an increase of the sternal metastasis infiltrating the surrounding soft tissues and an enlargement of liver metastases. The patient discontinued osimertinib, and, being not eligible for a systemic chemotherapy, died in April 2019.

## Discussion

TKIs significantly improved survival of *EGFR* mutated lung adenocarcinoma patients, but acquired resistance inevitably occurs. While on-target resistance mutations are more common with first/second generation TKIs (i.e. *EGFR* T790M), by-pass signaling pathways are prevalent with osimertinib (4). During treatment, multiple resistance mechanisms can arise simultaneously, and their detection can be challenging because of tumor heterogeneity, thus having important implications for treatment strategies. Herein, we presented an interesting case with the unusual coexistence of three specific resistance mechanisms, underlining the complementary role of both liquid and solid biopsy to monitor tumor dynamics. In the described case resistance to erlotinib was observed after 12 months of treatment and was associated with T790M mutation detected only on ctDNA, which disappeared under osimertinib treatment, according to previous published data (4). However, the tumor progressed, and a fine-needle aspiration of the sternal metastasis revealed the squamous cell transformation and *MET* amplification. The squamous lesion retained the sensitive *EGFR* mutation L858R and the *TP53* mutation S215R, suggesting the same clonal origin, without the T790M. The absence of T790M in the squamous lesion was confirmed also by digital droplet PCR. Considering that sternal lesion started progressing during the first line treatment, it is likely that both the squamous cell transformation and the *MET* amplification were already present, but not detectable, before osimertinib treatment. An important limitation of this case report is that it was not possible to determine *MET* status also on pleural effusion at erlotinib progression, because of the scarce available biological material.

Overall, the identification of alterations driving acquired resistance can provide patients with new therapeutic perspectives. For instance, MET inhibitors, such as savolitinib and tepotinib have been synthetized for patients with *EGFR* mutations that acquire resistance to TKI through *MET* alterations (5, 6). Unfortunately, the combined use of EGFR and MET TKIs was not considered because patient’s clinical conditions did not leave room for further lines of treatment after osimertinib failure.

Another interesting aspect is the increased PD-L1 expression, which was 10% at baseline and raised to 60% in the sternal metastasis during osimertinib treatment, confirming that some EGFR-TKIs resistant lung tumors can express high levels of PD-L1. It has been proven that this can be favored by some resistance mechanisms, such as *MET* amplification (7). Peng and collaborators demonstrated that *MET* amplification can cause an upregulation of PD-L1 expression and favors immune escape ability of *EGFR* mutant cancer cells (7). However, few studies have deeply examined the connection between EGFR TKI resistance and PD-L1 expression, and, consequently, the role of immunotherapy in this setting of patients is still unclear. The temporal and spatial tumor evolution is highly dynamic, making the management of EGFR-TKI resistant tumors challenging. Here it is described a case of EGFR-TKI resistance driven by T790M mutation with the rare co-occurrence of squamous cell transformation, *MET* amplification, and increased PD-L1 expression, mirroring the high degree of tumor heterogeneity induced by treatment pressure. In addition, a *TP53* mutation was detected: *TP53* alterations often co-occur with *EGFR* mutations and have a negative impact on TKI response and patient’s prognosis (8).

Despite the rare coexistence of multiple resistance mechanisms, response to first and second line EGFR TKIs was in good agreement with literature data (2, 3). Of course, squamous cell transformation is in itself associated with a poor prognosis and it is likely that MET amplification has greatly contributed to tumor progression.

## Data Availability Statement

The raw data supporting the conclusions of this article will be made available by the authors, without undue reservation.

## Author Contributions

All authors were involved in the clinical management of the presented case and participated in manuscript preparation. All authors contributed to the article and approved the submitted version.

## Conflict of Interest

The authors declare that the research was conducted in the absence of any commercial or financial relationships that could be construed as a potential conflict of interest.
